# Comparison of multiple algorithms to reliably detect structural variants in pears

**DOI:** 10.1186/s12864-020-6455-x

**Published:** 2020-01-20

**Authors:** Yueyuan Liu, Mingyue Zhang, Jieying Sun, Wenjing Chang, Manyi Sun, Shaoling Zhang, Jun Wu

**Affiliations:** Center of Pear Engineering Technology Research, State Key Laboratory of Crop Genetics and Germplasm Enhancement, Nanjing Agricultural University, Nanjing, 210095 Jiangsu China

**Keywords:** SV detection, NGS, Long-read sequencing, Sequencing depth, Accuracy of SVs, SV calling pipeline

## Abstract

**Background:**

Structural variations (SVs) have been reported to play an important role in genetic diversity and trait regulation. Many computer algorithms detecting SVs have recently been developed, but the use of multiple algorithms to detect high-confidence SVs has not been studied. The most suitable sequencing depth for detecting SVs in pear is also not known.

**Results:**

In this study, a pipeline to detect SVs using next-generation and long-read sequencing data was constructed. The performances of seven types of SV detection software using next-generation sequencing (NGS) data and two types of software using long-read sequencing data (SVIM and Sniffles), which are based on different algorithms, were compared. Of the nine software packages evaluated, SVIM identified the most SVs, and Sniffles detected SVs with the highest accuracy (> 90%). When the results from multiple SV detection tools were combined, the SVs identified by both MetaSV and IMR/DENOM, which use NGS data, were more accurate than those identified by both SVIM and Sniffles, with mean accuracies of 98.7 and 96.5%, respectively. The software packages using long-read sequencing data required fewer CPU cores and less memory and ran faster than those using NGS data. In addition, according to the performances of assembly-based algorithms using NGS data, we found that a sequencing depth of 50× is appropriate for detecting SVs in the pear genome.

**Conclusion:**

This study provides strong evidence that more than one SV detection software package, each based on a different algorithm, should be used to detect SVs with higher confidence, and that long-read sequencing data are better than NGS data for SV detection. The SV detection pipeline that we have established will facilitate the study of diversity in other crops.

## Background

Structural variants (SVs), which include deletions, insertions, inversions, duplications and translocations, are defined as rearrangements in chromosomes larger than 50 nucleotides [1]. Translocations can also be classified as intra-chromosomal translocations (ITXs) and inter-chromosomal translocations (CTXs), based on whether the chromosome of the source locus is the same as that of the target locus [2]. Deletions, insertions and duplications are called unbalanced SVs because they give rise to copy number variants (CNVs), while inversions and translocations are called balanced SVs [2]. It is clear that SVs play an important role in biological processes, and the identification of SVs is crucial for studying human genetic diversity, gene and genome variants, evolution and disease [3, 4]. SVs have been shown to be related to human diseases, such as immune escape of tumor cells [5], chronic hepatitis B virus infection [6] and heart failure [7]. SVs such as insertions and deletions and CNVs have been shown to contribute to natural variation of plants and have played a significant role in the differentiation of complex traits, domestication, evolution and adaptation [8, 9]. For example, a CNV involving four genes that define the *Female* locus in cucumber, which arose from a recent 30.2-kb duplication in a meiotically unstable region, gave rise to gynoecious plants [10]. The study of single nucleotide polymorphisms (SNPs), InDels and CNVs in tomato revealed introgressions from wild species and the mosaic structure of the genomes of cherry tomato accessions [11]. In ‘Su Shuai’ apple, SVs in 17 genes associated with disease resistance, 10 genes relevant to gibberellin and 19 genes related to fruit flavor were identified [12].

Pear is the third most important fruit species of the Rosaceae and is widely cultivated all over the world. The *Pyrus* genus is genetically diverse with thousands of cultivars, and studying SVs in *Pyrus* can lead to a better understanding of genetic diversity among cultivars and the genetic basis for complex traits. Previous studies have shown that SVs can influence crop traits, domestication, and evolution [8–12], but little is known about the SVs in *Pyrus*. Moreover, SV detection software was originally developed and tested using the human genome or the genome of the model plant *Arabidopsis thaliana*, so this software may not efficiently detect SVs in pear. Sequencing of the genome of *Pyrus bretschneideri cv.* ‘Dangshansuli’ pear, a variety that originated in China, in 2013 [13], revealed that it shows large differences from the *A. thaliana* genome. For example, the *A. thaliana* genome is smaller (only 125 Mb) and has fewer repetitive sequences than the genomes of pear and most fruit crops [13]. Thus, the development of a pipeline to detect SVs in *Pyrus* is of great significance for facilitating studies of genome complexity in the Rosaceae.

Recently, the availability of next-generation sequencing (NGS) and long-read sequencing data has greatly facilitated the characterization of SVs because variants of different sizes and types can be detected and breakpoints can accurately be identified at base-pair resolution [14–16]. NGS generates short reads ranging from 35 bp to 700 bp in length, while the long reads generated by third generation sequencing technology are over 10 kb in length [17]. A sufficient sequencing depth is required to detect SVs. For the human genome, 35-bp paired-end reads with an average depth of > 30× were used to build an accurate consensus sequence and characterize a million SNPs and 400,000 SVs [18]. A lower sequencing depth, > 10×, was found to be sufficient for detecting SVs when using reads over 10 kb in length [16]. However, the most suitable sequencing depth for detecting SVs in pear has not been determined.

To date, many approaches have been developed to detect SVs using NGS data. These algorithms are classified into four distinct categories based on the method used to detect SVs: read depth, read pairs, split reads, and assembly [19]. Algorithms based on read-depth signals can detect duplications and deletions using all mapped reads, but only at coarse resolution [20]. Read-depth algorithms are more effective for detecting larger (> 1 kb) CNVs. However, they cannot detect inversions. Read-pair algorithms are more popular for detecting SVs because of their relative simplicity and their ability to detect all SV types [21–23]. Split read-based callers can work with low-coverage NGS data and identify SVs with base resolution. However, the disadvantages of split-read callers are that they cannot detect larger SVs such as duplications, inversions, translocations, and more complex variants because some short reads may map to many locations in the reference genome [24, 25]. When using assembly-based callers (de novo and reference-based assembly callers), short reads need to be assembled into longer sequence stretches called contigs before detection [26]. Because the contigs are longer than individual reads, SVs are called with high confidence. Many software packages have been developed for detecting more types of SVs with higher accuracy by integrating multiple algorithms (such as DELLY [27] and Lumpy [28]) or merging the outputs of multiple software (such as FusorSV [29], MetaSV [30] and Parliament2 (https://github.com/dnanexus/parliament2)). Callers using NGS data have a high rate of SV miscalling due to errors in alignment or de novo assembly, especially in repetitive regions that cannot be spanned with short reads [31]. To overcome these issues, software using long-reads such as SVIM [32] and Sniffles [16] have been developed; these algorithms are mostly based on split reads. The functions and features of each type of SV-calling software are known, but the reliability of using different combinations of software for detecting SVs has not been studied.

In this paper, we evaluated the effectiveness of several types of SV detection software in *Pyrus*. The pear cultivar chosen was ‘Yali’ (*P. bretschneideri*), which is genetically closely related to ‘Dangshansuli’ (*P. bretschneideri*) and is one of the primary pear cultivars grown in China. This cultivar is also exported to other countries where it is known as Asian pear. We have conducted a systematic analysis using ‘Yali’ genome NGS and long-read sequencing data to compare the performances of several commonly used SV-calling software packages using short reads, namely Pindel [25], BreakDancer [33], IMR/DENOM [34], Platypus [35], DELLY [27], Lumpy [28], and MetaSV [30], and software packages using long reads, namely SVIM [32] and Sniffles [16]. The effects of different sequencing depths on SV detection were investigated, and the most appropriate sequencing depth for detecting SVs in *Pyrus* was determined by comparing the number of SVs detected and the computational resources required for different sequencing depths. Moreover, we investigated the overlap in SVs identified by all possible combinations of two or three software packages to obtain high-confidence SVs. Then, the reliability of selected ‘Yali’ pear SVs was verified using visualization tools. Our findings lay the foundation for subsequent studies of SVs, and the pipeline we constructed can be used to reliably detect SVs in other crops.

## Results

### Sequencing and mapping of the ‘Yali’ genome

Short read sequencing of the pear ‘Yali’ genome was conducted using the IIIumina HiSeq™ 2000 platform for pair-end sequencing, and the sequencing depth was 60×. A total of 103,584,796,150-bp reads were obtained, and the GC content was 39%. The quality of the raw resequencing data was determined using FastQC (https://www.bioinformatics.babraham.ac.uk/projects/fastqc/) software. After using Trimmomatic [36] to filter the low quality sequencing data, 97.84% of the reads were kept. Of the clean reads, 97.15% were mapped to the ‘Dangshansuli’ pear genome using Burrows-Wheeler-Aligner (BWA) software [37]. Seven SV detection software packages using NGS data (Table 1) were then used to identify SVs in ‘Yali’.

**Table 1:** Comparison of the nine types of SV detection software

Data type	Detection tools	Detectable SV types								Algorithms
		INS	DEL	INV	DUP	ITX	CTX	TRA	MNPs	
Illumina data	Pindel	Yes	Yes	Yes	Yes	No	No	No	No	SR
	BreakDancer	Yes	Yes	Yes	No	Yes	Yes	No	No	RP
	DELLY	Yes	Yes	Yes	Yes	No	No	Yes	No	RP + SR
	IMR/DENOM	Yes	Yes	No	No	No	No	No	No	AS
	LUMPY	No	Yes	Yes	Yes	No	No	Yes	No	RP + SR
	Platypus	Yes	Yes	No	No	No	No	No	Yes	AS
	MetaSV	Yes	Yes	Yes	Yes	Yes	Yes	Yes	No	–
PacBio data	Sniffles	Yes	Yes	Yes	Yes	No	No	Yes	No	SR
	SVIM	Yes	Yes	Yes	Yes	No	No	No	No	SR

Notes. An overview of the nine SV callers, including the types of SVs detected (INS: insertion, DEL: deletion, INV: inversion, DUP: duplication, TRA: Translocation, ITX: intra-chromosomal translocation, CTX: inter-chromosomal translocation) and the mutation signals used (SR: split reads, RP: read pairs, AS: assembly). The symbol ‘-’ indicates that the algorithm is chosen by the user

Long-read sequencing data for ‘Yali’ were generated using the PacBio platform, and the sequencing depth was 30×. A total of 2,977,899 subreads were obtained. The average subread length was 6 kb and the N50 was 8 kb. Two SV detection software packages (Sniffles and SVIM) using long read sequencing data (Table 1) were selected to identify SVs in ‘Yali’.

### SVs between ‘Yali’ and the reference genome detected using different algorithms and sequencing data

**Fig. 1:**
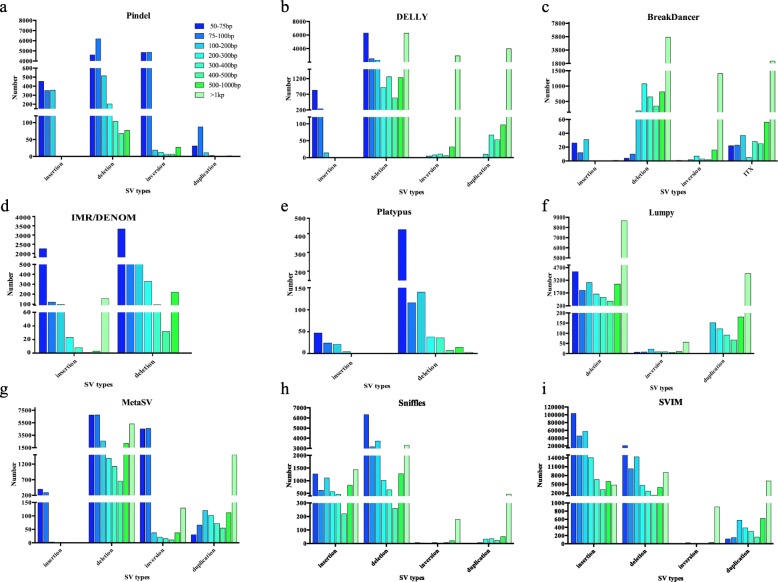
The number and types of SVs were called by seven software packages (Pindel, DELLY, BreakDancer, IMR/DENOM, Platypus, Lumpy, MetaSV) using next-generation sequencing data (60× sequencing depth), and two software packages (Sniffles, SVIM) applied long-read sequencing data (30× sequencing depth). The panel labels in Pindel (**a**) are also applied to DELLY (**b**), BreakDancer (**c**), IMR/DENOM (**d**), Platypus (**e**), Lumpy (**f**), MetaSV (**g**), Sniffles (**h**), SVIM (**i**)

Depending on the performances of the nine SV callers, which are based on different algorithms (Table 1), up to eight types of SVs in the ‘Yali’ genome were detected: insertions, deletions, inversions, duplications, translocations, MNPs (multiple nucleotide polymorphisms), CTXs and ITXs (Table 1). Deletions were the only SVs detected by all nine callers. The number of SVs detected by the nine callers, categorized based on type and length, is shown Fig. 1. Of the nine SV callers, SVIM detected the highest number of SVs. The software with assembly-based algorithms called fewer SVs than the other types of software, and Platypus called the fewest SVs. Although both DELLY and Lumpy use split-read and read-pair algorithms, DELLY called a higher number of SVs and more types of SVs than Lumpy. Detailed information about the number of SVs called by each software package is shown in Fig. 1.

For Pindel, which uses an split-read algorithm, short reads need to be broken into smaller fragments and mapped separately to the reference genome [25]. A total of 22,548 SVs were found using Pindel: 1178 insertions, 11,445 deletions, 9791 inversions and 134 duplications (Fig. 1). Deletions accounted for the largest proportion (50.76%) of the SVs and inversions accounted for the second largest proportion (43.42%). Compared with deletions and inversions, the numbers of insertions and duplications were very small, accounting for 5.22 and 0.59% of the SVs, respectively. In addition, Pindel could not detect insertions greater than 200 bp in length in the ‘Yali’ pear genome. Therefore, Pindel performed better in detecting small insertions and deletions and only detected a limited number of large SVs (>l kb) (Fig. 1).

BreakDancer detects SVs using a read-pair algorithm; reads that map with an abnormal insert size or orientation are collected and then classified as insertions, deletions, inversions, or translocations [33]. Using BreakDancer, a total of 8682 SVs were detected: 90 insertions, 6900 deletions, 1398 inversions, and 294 ITXs. Of the SVs 79.47% were deletions, and no insertions longer than 400 bp were identified (Fig. 1). Therefore, BreakDancer is not suitable for detecting small variants or large insertions in pear.

IMR/DENOM [34] utilizes local de novo assemblies and iterative read mapping to the reference sequence to identify SVs [38]. IMR/DENOM called a total of 8398 SVs (2514 insertions, 5884 deletions). IMR/DENOM could detect large insertions (> 1 kb) but it could not detect large deletions in ‘Yali’ (> 1 kb) (Fig. 1).

Platypus [35] detects deletions and insertions when using the assembly option, but this caller detected fewer and smaller SVs than the other callers; only 92 insertions, 776 deletions and 886 other complex SVs were detected. Moreover, Platypus could not call insertions longer than 300 bp, and over 50% of the SVs identified ranged from 50 bp to 75 bp in length. Therefore, this software performed better in detecting small insertions and deletions (Fig. 1).

DELLY has the ability to integrate pair-end data from libraries with different insert sizes with split-read data, making it a versatile tool for analyzing SVs using deep whole-genome sequencing data [27]. Using DELLY, 1054 insertions, 20,991 deletions, 2976 inversions and 4217 duplications were identified (Fig. 1). About 30% of deletions were longer than 1 kb. Similar to Pindel, DELLY could not detect insertions longer than 200 bp. However, unlike Pindel, DELLY was not capable of detecting inversions and duplications less than 100 bp in length. Moreover, more than 97% of the inversions and more than 94% of the duplications called by DELLY were greater than 1 kb in length.

Lumpy [28] integrates multiple algorithms including those using read pairs, split reads and read depth. It detected 24,072 deletions, 127 inversions, and 4620 duplications. Over 35% of deletions, 44% of inversions and 87% of duplications were longer than 1 kb (Fig. 1). Therefore, Lumpy has superior sensitivity in detecting SVs longer than 1 kb.

MetaSV [30] detects SVs by merging the outputs of other SV detectors, such as Pindel, BreakDancer and Lumpy. It can also detect insertions by analyzing soft-clipped reads from alignments and improve the breakpoints of SVs using local assembly. To further compare the accuracy of SVs called by Pindel, BreakDancer and Lumpy, we only used the merge option without soft-clip-based analysis or local assembly. According to the merged results, 689 insertions, 26,770 deletions, 9381 inversions and 2057 duplications were detected (Fig. 1). Almost all insertions and inversions ranged from 50 bp to 100 bp in size, and over 50% of deletions were between 50 bp and 100 bp in length. More than 50% of duplications were longer than 1 kb.

Sniffles, which uses long-read sequencing data [16], detects SVs from long-read alignments using a split-read algorithm with the NGMLR aligner. It detected 6556 insertions, 19,774 deletions, 242 inversions and 633 duplications (Fig. 1). The other software package using long-read sequencing data, SVIM [32], detects SVs in a process consisting of three steps: collection, clustering and combining of SVs from read alignments. SVIM detected 242,429 insertions, 67,950 deletions, 1019 inversions and 8609 duplications. SVIM detected more SVs than Sniffles, suggesting that SVIM detects SVs with higher sensitivity (Fig. 1).

### The SVs identified by multiple software are more accurate

We next investigated the overlap between SVs detected by multiple SV callers that use NGS data (each based on a different algorithm). The Integrative Genomics Viewer (IGV) browser was first used to confirm the presence of the SVs called by each caller. We randomly selected 660 deletions ranging from 50 bp to 500 bp in length from the output of single callers using NGS data. The accuracies of each type of software are shown in Additional file 8. The accuracies of Pindel (58%) and BreakDancer (58%) were lower than those of the other callers. For Pindel, the accuracy in calling SVs ranging from 50 bp to 75 bp in size was 75% while the accuracy in calling SVs ranging from 400 bp to 500 bp in size was 33%. Therefore, Pindel detected small SVs with high sensitivity and confidence, with accuracy decreasing as SV length increased. The DELLY and Lumpy algorithms performed similarly, and the accuracy of SVs called by DELLY (63%) was a little better than that of Lumpy (60%). For the IMR/DENOM and Platypus software packages, which are based on assembly, the average accuracies of SV detection (81 and 66%, respectively) were higher than those of the other types of software, demonstrating that callers based on assembly algorithms detect SVs with higher confidence. The accuracy of the SVs called by MetaSV (70%), which were merged from the results of Pindel, BreakDancer and Lumpy, was higher than that of each caller alone. Therefore, the SVs called by merging outputs from multiple callers are more accurate than single SV caller.

According to the performances of the seven software packages using NGS data, Pindel, BreakDancer, IMR/DENOM and DELLY were selected for finding overlapping SVs (Table 2). Because the SVs called by MetaSV were merged from the outputs of Pindel, BreakDancer and Lumpy, we simply combined the outputs of MetaSV and IMR/DENOM to identify overlapping SVs and determine whether they were more accurate. We found the number of overlapping SVs from random combinations of Pindel, BreakDancer, IMR/DENOM and DELLY (Table 2). Based on the percentages of overlapping insertions, deletions, inversions and duplications identified by each software, DELLY performed better than the other three software packages (Table 2).

**Table 2:** The number of structural variations detected by individual algorithms and combinations of algorithms

Combination	Insertion	Deletion	Inversion	Duplication
Pindel	1178	11,445	9791	134
DELLY	1054	20,991	2976	4217
BreakDancer	90	6900	1398	0
IMR/DENOM	2514	5884	0	0
Pindel-DELLY	3	8782	7997	89
Pindel-BreakDancer	0	7616	6442	0
Pindel-IMR/DENOM	1	502	0	0
DELLY-BreakDancer	0	1192	129	0
DELLY-IMR/DENOM	307	5152	0	0
BreakDancer-IMR/DENOM	0	4729	0	0
Pindel-DELLY-IMR/DENOM	1	443	0	0
Pindel-DELLY-BreakDancer	0	7613	6441	0
DELLY-BreakDancer-IMR/DENOM	0	4423	0	0
Pindel-BreakDancer-IMR/DENOM	0	361	0	0

When focusing on Pindel and DELLY, we found very little overlap in the insertions identified by the two programs, with only 0.25% of Pindel insertions and 0.28% of DELLY insertions overlapping. However, greater than 80% of inversions were predicted by both software. A high percentage, 66.42%, of the duplications identified by Pindel were also identified by DELLY, but only 2.11% of those identified by DELLY were also identified by Pindel. There was a higher number of overlapping deletions, with 76.73% of Pindel deletions also identified by DELLY, and 41.83% of DELLY deletions identified by Pindel.

The number of overlapping SVs between IMR/DENOM and Pindel and between IMR/DENOM and DELLY were shown in Table 2, respectively. Since IMR/DENOM can only detect insertions and deletions (Table 1), the number of inversions and duplications overlapping with those identified by the other three software packages was 0. Only one insertion and 502 deletions were detected by both Pindel and IMR/DENOM. Of the deletions identified by IMR/DENOM, 8.53% were also identified by Pindel, and 66.54% of the Pindel deletions overlapped with the IMR/DENOM deletions. For IMR/DENOM and DELLY, 307 insertions and 5152 deletions were discovered by both programs. Of the DELLY insertions, 26.06% were identified by IMR/DENOM, and 12.21% of IMR/DENOM insertions were identified by DELLY. However, 45.02% of the DELLY deletions overlapped with those identified by IMR/DENOM, while over 85% of IMR/DENOM deletions were identified by DELLY. IMR/DENOM and BreakDancer had no overlapping insertions, while the number of overlapping deletions was 4729.

There were few overlapping insertions between BreakDancer and DELLY and between BreakDancer and Pindel. However, a large number of deletions were called by both BreakDancer (100% overlapped with Pindel deletions) and Pindel (66.54% overlapped with BreakDancer deletions). Although 100% of the BreakDancer deletions also overlapped with those identified by DELLY, only 5.68% of DELLY deletions were identified by BreakDancer.

When comparing the combination of three software packages, few of the insertions called by Pindel, DELLY and IMR/DENOM overlapped, and no insertions called by these programs overlapped with those called by BreakDancer. However, there was better overlap in the deletions called by combinations of three software. Although Pindel, DELLY and IMR/DENOM shared fewer than 10% of deletions with each other, when comparing the output of Pindel, DELLY and BreakDancer, all of the deletions identified by BreakDancer, 66% of the deletions identified by Pindel and 36.27% of deletions identified by DELLY overlapped. A high number of overlapping inversions was also observed when combining DELLY (100%), BreakDancer (100%) and Pindel (65.78%). When comparing DELLY, BreakDancer and IMR/DENOM, 21.07% of deletions identified by DELLY, 75.17% of those identified by IMR/DENOM and 64.10% of those identified by BreakDancer overlapped. When comparing Pindel, IMR/DENOM and BreakDancer, 3.16% of deletions identified by Pindel, 5.23% of those identified by BreakDancer and 6.14% of those identified by IMR/DENOM overlapped.

To confirm the accuracy of SVs from multiple software packages using NGS data, we randomly chose 940 overlapping SVs from the output of two software packages combined and three packages combined. The average accuracy of overlapping deletions was higher than the accuracy of deletions called by a single software package (Additional file 8). Moreover, the accuracies of SVs identified by the combinations Pindel and DELLY, Pindel and BreakDancer, and DELLY and BreakDancer were lower than those of SVs identified by the combinations Pindel and IMR/DENOM, DELLY and IMR/DENOM, and BreakDancer and IMR/DENOM. The average accuracy of overlapping SVs identified by Pindel, DELLY and BreakDancer was lower than that of overlapping SVs identified by Pindel, DELLY and IMR/DENOM; DELLY, BreakDancer and IMR/DENOM; and Pindel, BreakDancer and IMR/DENOM. In particular, the average accuracy of overlapping deletions from MetaSV, which included the merged results of Pindel, BreakDancer and Lumpy, and IMR/DENOM was greater than 90%. This indicates that the SVs detected by a combination of assembly-based software and multiple algorithm-based software were more accurate than those detected by the other combinations of software.

To further validate the accuracy by long-read resequencing data, we randomly selected 300 SVs identified by the software packages from Sniffles (100 SVs), SVIM (l00 SVs) and Sniffles_SVIM (SVs). The average accuracy of SVs detected by Sniffles was greater than 95%, while the accuracy of SVs detected by SVIM was less than 80%. The SVs overlapping between Sniffles and SVIM were high confidence SVs with an accuracy greater than 96%. Compared with algorithms using NGS data, the algorithms using long-read sequencing data detected SVs with higher accuracy, and large SVs with more confidence. However, the SVs overlapping between MetaSV and IMR/DENOM were more accurate than those overlapping between Sniffles and SVIM, which suggests that SVs detected by a combination of assembly-based software and multiple algorithm-based software are the most accurate.

**Fig. 2:**
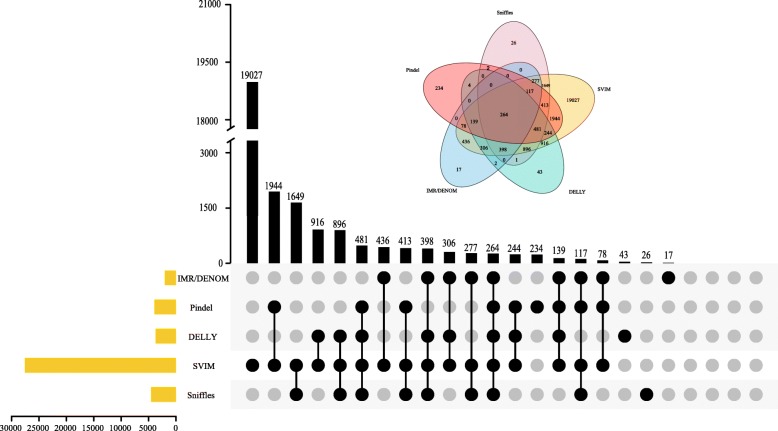
Comparison of the number of genes within SVs identified using NGS-based software and long-read sequencing-based software. The yellow bars indicate the number of SVs identified by an individual software package and the black bars indicate the number of SVs identified by combinations of software packages

We then annotated the SVs detected by five individual callers, three using NGS data, each based on a different algorithm (Pindel, DELLY, and IMR/DENOM), and two using long-read sequencing data (Sniffles, which detected more SVs, and SVIM, which detected higher-confidence SVs), and observed the number of genes within SVs commonly identified by these callers (Fig. 2). Among the callers based on paired-read algorithms, DELLY was chosen because it performed better than BreakDancer and Lumpy. The assembly-based caller IMR/DENOM was chosen because it detected more SVs than Platypus. The split-read-based caller, Pindel was chosen because it was better able to detect SVs less than 100 bp in length. A total of 264 genes within SVs were detected using the five software packages. These genes were subjected to functional enrichment analysis using both the GO (Gene Ontology) and KEGG (Kyoto Encyclopedia of Genes and Genomes) databases (results are shown in Additional files 1 and 2). These 264 genes will be the main targets for future functional studies of the variants between ‘Yali’ and ‘Dangshansuli’ pear. A total of 403 genes within SVs were commonly detected by the callers using NGS data, and 4495 genes within SVs were commonly detected by the callers using long-read sequencing data (Additional file 3: Figure S1(a) and (b), respectively). The results of GO and KEGG analysis of these genes are shown in Additional files 4, 5, 6 and 7.

### Effect of sequencing depth on SV detection

**Fig. 3:**
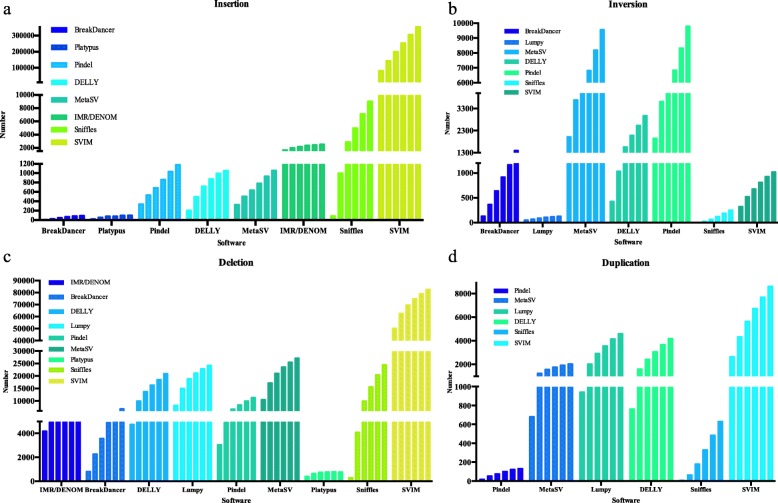
The number of four SV types ( Insertion (**a**), Inversion (**b**), Deletion (**c**), Duplication (**d**)) were identified by nine software packages at different sequencing depths. There are six bars for each software, and each bar indicates the number of variants identified at the different sequencing depth. The sequencing depths for software using NGS data are 10×, 20×, 30×, 40×, 50× and 60×, and those for software using long-read sequencing data are 5×, 10×, 15×, 20×, 25×, and 30×

To determine the most appropriate sequencing depth for detecting SVs in pear, the performances of all software packages using NGS data (except MetaSV) and both software packages using long-read sequencing data at different sequencing depths were compared. *Seqtk* was used to obtain NGS (10×, 20×, 30×, 40×, 50×, 60×) and long-read sequencing (5×, 10×, 15×, 20×, 25×, 30×) data at different sequencing depths (Fig. 3). For IMR/DENOM and Platypus, the number of SVs increased as sequencing depth increased to 50×. When the NGS depth increased to 60×, the number of variants called by IMR/DENOM and Platypus did not change too much, and even decreased. Based on this analysis, for assembly-based software an NGS depth of 50× is sufficient for detecting SVs in *Pyrus*. For Pindel, BreakDancer, DELLY, Lumpy, Sniffles and SVIM, the number of SVs called obviously increased as the sequencing depth increased. Therefore, for split read-based and read pair-based software, the higher the depth of sequencing, the higher the number of SVs detected in *Pyrus*.

**Fig. 4:**
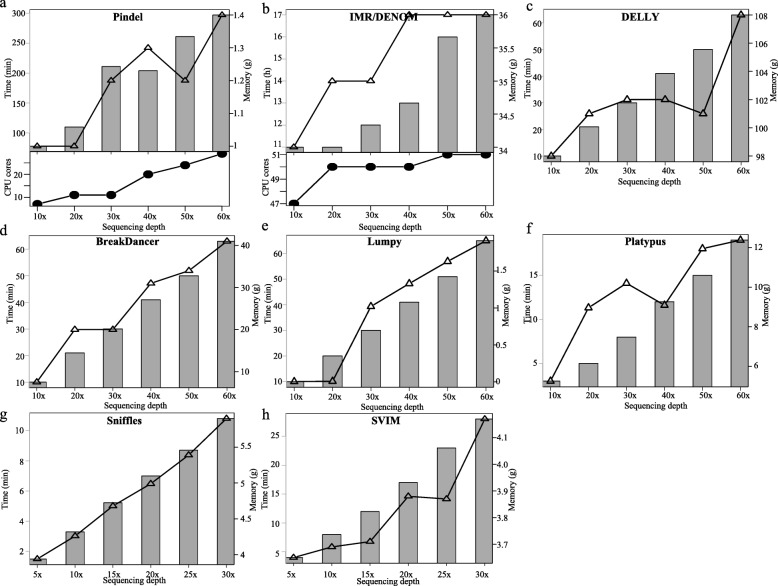
Effect of sequencing depth on the running efficiency of eight types of software (Pindel (**a**), IMR/DENOM (**b**), DELLY (**c**), BreakDancer (**d**), Lumpy (**e**), Platypus (**f**), Sniffles (**g**), SVIM (**h**)). The cost of time, CPU cores and memory were compared at different sequencing depth

The computational time, the number of CPU cores required, and memory cost also need to be considered when determining the most suitable sequencing depth. Therefore, software performance at different sequencing depths was also evaluated. The performance of each SV caller was determined based on the mean computational time and computational memory cost with different parameters. The running time and maximum memory occupancies for the eight callers at different sequencing depths are shown in Fig. 4. When running DELLY, BreakDancer, Lumpy and SVIM, threads cannot be set, so the default CPU core was one. However, for Pindel, IMR/DENOM and Sniffles, different threads can be set to decrease the computational time for SV detection depending on the running environment. Therefore, we set the thread to 50 for these programs to improve the detection efficiency. The number of CPU cores for Platypus can be specified, and we used 50 CPU cores. Platypus was able to detect SVs much faster than IMR/DENOM; Platypus required only about 3 min while IMR/DENOM required more than 10 h. As the depth of sequencing increased, so did the computational time, memory and the number of CPU cores required (Fig. 4). IMR/DENOM required more CPU cores and memory than the other programs. Sniffles was faster than SVIM, and both programs required the same amount memory. To sum up, for NGS data, DELLY is recommended because it requires less computational time and memory and because combinations of software that include DELLY identify more overlapping SVs than those that do not. If enough CPU cores and free memory on the server machine are available, IMR/DENOM is more suitable because of its high sensitivity and accuracy in detecting SVs. For long-read sequencing data, both Sniffles and SVIM are recommended, since SVIM can detect more SVs and Sniffles detects SVs with high confidence.

### Workflow for detecting accurate SVs

**Fig. 5:**
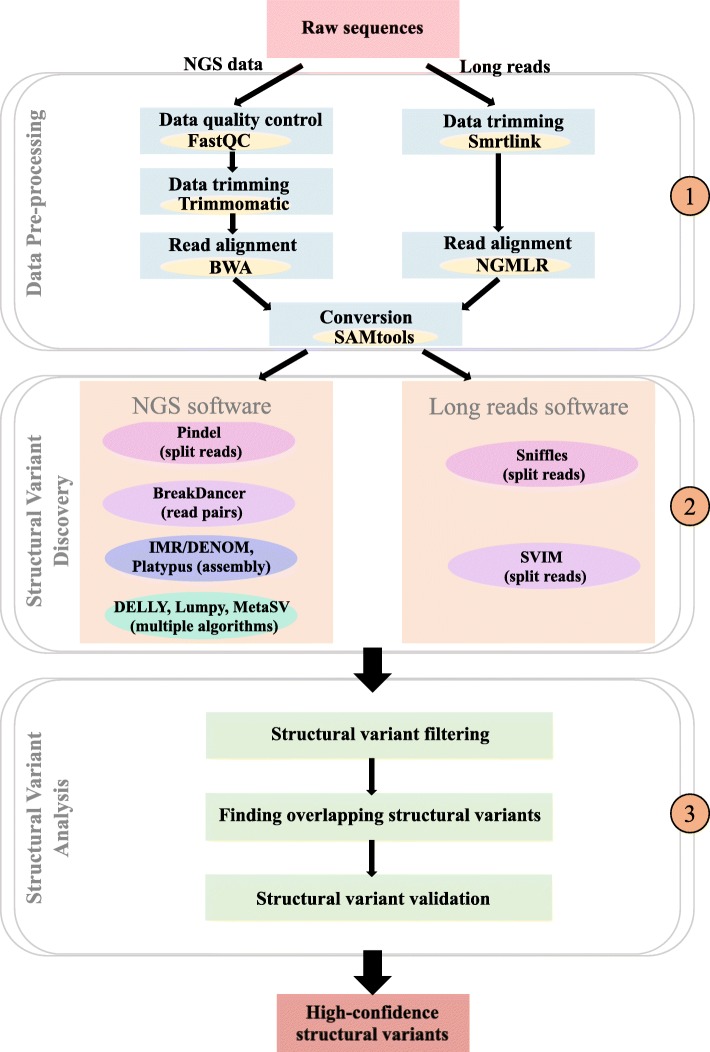
The overall workflow for SV detection based on four algorithms using NGS and long-read sequencing data

The goal of this study was to detect SVs with higher accuracy using the ‘Yali’ resequencing data. To facilitate the study of SVs in the future, we set up a workflow for SV detection based on the different algorithms evaluated in this study (Fig. 5). The workflows for SV detection using NGS data and long-read sequencing data were similar. Therefore, we describe the workflow for NGS data as an example. Firstly, quality control of the raw resequencing data was done, trimming the reads to obtain clean reads. Secondly, we mapped the clean reads to the ‘Dangshansuli’ reference genome. Thirdly, nine SV-calling software were used to detect SVs, and the overlapping SVs were identified using multiple types of software. The seven software packages using NGS data were mainly classified into two categories: software based on a single algorithm and software based on multiple algorithms. Pindel uses split reads and BreakDancer uses read pairs. IMR/DENOM and Platypus are based on assembly. The algorithms of Lumpy and DELLY are similar and both use read pairs and split reads. The algorithms of MetaSV merge outputs from multiple software. The overlapping SVs identified by multiple software packages were more accurate. This high accuracy is essential when selecting SVs for further study.

## Discussion

In our study, we compared the sensitivities, accuracies and computational equipment requirements of seven common software packages using ‘Yali’ pear NGS data and two software packages using ‘Yali’ pear long-read sequencing data to provide insights for choosing the most appropriate SV-calling program. Detecting more SV types and decreasing the false discovery rate and increasing the sensitivity of SV detection have always been a concern for researchers studying SVs. Software developers have also focused on improving the sensitivity of SV detectors [27–29]. Here, we focused on the performance of seven software packages using NGS data (Fig. 1) and found that each software has its own advantages and disadvantages. For example, MetaSV detected many more SVs than the other packages tested; however, before running MetaSV, the VCF (variant call format) files of Pindel, BreakDancer, Lumpy or other software need to be prepared, which is a cumbersome process. Not all software can detect insertions, and all programs except IMR/DENOM had limitations in the length of insertions detected. Using SAMtools, the mean sequence insert size of ‘Yali’ was found to be 320 bp. Only IMR/DENOM can detect insertions > 300 bp (Fig. 1), which explains why IMR/DENOM was the only caller able to detect insertions longer than the sequence inserts. Breakdancer, DELLY, Lumpy and MetaSV were more sensitive in detecting large deletions and Pindel was more sensitive in detecting small SVs (Fig. 1); this is because read-pair algorithms are less sensitive in detecting small SVs, which are below the standard deviation for insert size [14, 25, 27, 28]. Of the two software packages using long-read sequencing data, SVIM showed higher sensitivity, probably because SVIM collects, clusters and combines SV signatures from read alignments [32]. By contrast, Sniffles detects SVs from analysis of split-read alignments, high-mismatch regions and sequencing depth and coverage [16].

Validating the presence of SVs has been a challenge for researchers using the pear genome, but progress has been made in other model systems. For the human genome, a map of SVs was constructed based on whole-genome sequencing data from 185 human genomes, and most SVs were mapped to nucleotide resolution [39]. Many types of SV-calling software have been developed, and the sensitivities and false discovery rates of these callers have been tested using known SVs [28, 29]. In maize and rice, SVs were identified and mapped using the results of pan-genome analyses of population structure and diversity [40, 41]. However, the SVs in pear populations are unknown and characterizing high-confidence SVs is crucial for studying SVs using a single sample of pear. To evaluate the accuracy of different SV callers, we first compared the accuracy of SVs called by each software package, then selected five software packages for finding overlapping SVs, and finally validated the accuracy of the overlapping SVs. The overlapping SVs from multiple packages had higher confidence then single software package.

Sequencing costs are often the biggest limitation for many laboratories [42]. For single nucleotide variant and small InDel detection, an average sequencing depth of 30× is the standard [43, 44]. But for SV detection, sensitivity and breakpoint detection can improve with increasing sequencing depth [42]. In our study, we found that the depth of NGS and long read sequencing absolutely affected the number of SVs called (Fig. 3), the cost of sequencing (Fig. 4) and the speed of variant calling (Fig. 4). Increasing sequencing depth within a certain range can increase the number of SVs identified and reduce the false discovery rate of variant calling when using NGS data. For IMR/DENOM and Platypus, the number of SVs called did not change with sequencing depths greater than 50×, and the computational time, number of CPU cores and memory required also showed no remarkable change. Therefore, when using IMR/DENOM and Platypus to detect SVs, the appropriate sequencing depth is 50×. By contrast, we did not identify an optimal sequencing depth for Pindel, DELLY, BreakDancer, Lumpy, MetaSV or software using long-read sequencing data (Sniffles and SVIM). For NGS-based callers with split-read, read-pair and read-depth algorithms and long-read sequencing-based callers with split-read algorithm, as the sequencing depth increased, the number of SVs called and the computational time, number of CPU cores and memory needed also increased.

## Conclusions

An SV detection pipeline using NGS and long-read sequencing data has been developed for application in pear, and this pipeline can be used for the study of SVs in other crops. Seven different types of SV-calling software packages using NGS data and two packages using long-read sequencing data were compared. SVIM detected SVs with the highest sensitivity, and Sniffles called SVs with the highest confidence. The SVs detected by software packages using long-read sequencing data showed higher accuracy than those using NGS data and also required less time, fewer CPU cores and less memory. A combination of multiple software packages is recommended for the detection of more types of SVs with higher accuracy. For NGS data, a sequencing depth of 50× is the most suitable for detecting SVs in pear based on the performance of assembly algorithm-based software. This information about the accuracy of SV detection, computational equipment requirements and suitable sequencing depth will benefit researchers engaged in the study of SVs. This study has provided important insights into methods for improving SV detection that can be applied in future studies of crop genomes.

## Methods

### Pear accession sequencing

‘Yali’ plants were grown in an experimental nursery at Changli Fruit Research Institute, Hebei Academy of Agricultural Sciences, China. Young leaves were collected 15 days after flowering, and extraction of DNA for NGS was performed using the Qiagen DNeasy 96 Plant Kit (Cat. no. 69181) following the manufacturer’s protocol. Paired-end sequencing libraries with an insert size of approximately 350 bp were sequenced on the Illumina HiSeq™ 2000 platform at the Biomarker Technologies Company (Beijing, China).

Long-read sequencing data have high error rates: ~ 15% with PacBio sequencing, and as high as ~ 40% with Oxford Nanopore sequencing [45]. We selected PacBio sequencing technology, and libraries for PacBio genome sequencing were constructed following the standard protocols from Pacific Biosciences. In brief, high molecular weight genomic DNA was sheared to a target size of 20 kb, followed by damage repair and end repair, blunt-end adaptor ligation, and size selection. Finally, the libraries were sequenced on the PacBio Sequel platform.

### Quality control of NGS sequencing data

*FastQC* was used to check raw sequencing data in FASTQ format during the first major step of sequence data pre-processing (fastqc -o yali_fastq yali_1.fq yali_2.fq). Based on the FastQC results, the overall quality values of raw sequence reads were calculated and reported in FASTQ format [46]. Trimmomatic, which is a fast, multithreaded command line tool for trimming paired-end and single reads produced by Illumina NGS technology [36], was used to trim reads using the following parameters: java –jar trimmomatic-0.36.jar PE -phred33 -trimlog logfile yali_1.fq yali_2.fq yali.read_1.fq yali.trim.read_1.fq yali.read_2.fq yali.trim.read_2.fq ILLUMINACLIP: /Trimmomatic/adapters/TruSeq3-PE.fa:2:30:10 LEADING:3 TRAILING:3 SLIDINGWINDOW:4:15 MINLEN:36. The output of Trimmomatic was in the form of uncompressed filtered FASTQ files.

### Read mapping

Most SV detection software packages using NGS data detect SVs from alignments generated using the BWA aligner. However, the software packages using long-read sequencing data have higher requirements for aligners. Many new aligners have been developed for long-read alignment. NGMLR is recommended because of its better performance compared with other aligners [16]. For NGS data, once the sequences were quality checked and trimmed, the next step was to align the sequences to the ‘Dangshansuli’ pear genome (http://peargenome.njau.edu.cn/). Firstly, the reference genome fasta file was indexed using BWA (bwa index dangshansuli.fasta). The pair-end sequencing reads of ‘Yali’ were aligned to the ‘Dangshansuli’ reference genome using the ‘align’ step (bwa aln -t 20 dangshansuli.fasta yali.read_1.fq > yali.read_1.sai; bwa aln -t 20 reference yali.read_2.fq > yali.read_2.sai; bwa sampe dangshansuli.fasta yali.read_1.sai yali.read_2.sai yali.read_1.fq yali.read_2.fq > yali.sam). For PacBio sequencing data, Smrtlink v8.0 was used to filter low quality raw data and processed long reads with accuracies higher than 0.8 (https://www.pacb.com/support/software-downloads/). NGMLR was used to map long reads to the ‘Dangshansuli’ reference genome (ngmlr –t 50 –r dangshansuli.fasta -q yali_pacbio.fastq –o yali.sam). Both the BWA and NGMLR results were in SAM format. SAMtools was used to convert SAM files into BAM files and to sort the BAM files and remove duplicates [47].

### Randomly extracting data at different sequencing depths

*Seqtk* can help users to process sequences in FASTA or FASTQ format (https://github.com/lh3/seqtk). The command ‘seqtk sample’ was used to randomly extract a subsample of reads from the clean reads. The short reads were sampled at depths of 10×, 20×, 30×, 40×, 50× and 60×. The long reads were sampled at depths of 5×, 10×, 15×, 20×, 25× and 30 × .

### Description of the four types of SV software

The next step after mapping reads to the reference genome was to identify the SVs from the processed BAM format files. Seven types of SV-calling software using NGS data and two types of SV-calling software using long-read sequencing data, each based on different algorithms, were used to detect the SVs between ‘Yali’ and ‘Dangshansuli’ pear.

#### Pindel [25]

Pindel v0.2.5b9 is a C++ application based on the SR algorithm. Running Pindel requires two steps. The first step is ‘bam2pindel.pl’, the purpose of which is to extract read pairs for use by Pindel (bam2pindel_bwa.pl -i yalisortrmdup.bam -o output_prefix -s yali –om). The second step is ‘pindel’; the input files were the ‘bam2pindel.pl’ file and the reference fasta file (Pindel –f dangshansuli.fasta –p bam2pindel.txt –o output –T 50). By default, Pindel detects all chromosomes if the chromosome region is not specified. Pindel can identify the break points of large deletions (1 bp-10 kb) and medium-sized insertions (1–20 bp) from paired-end short reads. The output file contains the type and size of SV, the chromosome ID, the break point coordinates and the number of reads supporting each event.

#### BreakDancer [33]

BreakDancer-max v1.4.5 is a Perl application and is based on read pairs. The software includes two complementary programs, ‘bam2cfg.pl’ (bam2cfg.pl -g –h yalisortrmdup.bam > config_file) and ‘breakdancer-max’ (breakdancer-max -lh config_file > output). The ‘bam2cfg.pl’ program is aimed at converting the BAM file into the specific file needed for ‘breakdancer-max’. The option ‘-l’ was chosen to analyze the Illumina long insert (mate-pair) library. The output contained important information such as the type and size of SV, the chromosome ID, and the SV length. BreakDancer-max had the ability to predict five types of SVs from ‘Yali’ sequencing data: insertions, deletions, inversions, and inter- and intra-chromosomal translocations.

#### IMR-DENOM [34]

IMR/DENOM v0.4.0 comprises three independent programs, namely ‘IMR’, ‘DENOM’, and ‘MCMERGE’, and is based on assembly. ‘IMR’ is designed to iterate realignment to the reference genome. In brief, in each iteration, reads are aligned to the reference genome and high-confidence SNPs and InDels are called and incorporated into a new consensus.

Before running the program, the config file was prepared, which contained the path of the output folder, reference, loaddata and settings for the iterations and threads. ‘imr easyrun’ (imr easyrun –e imr.bam –imrnocall configfile) produces the sample BAM file, and the input file was configfile. The option ‘-e’ was set to specify the bam file and ‘--imrnocall’ was chosen to only map reads and merge bam files without calling variants. Moreover, ‘-p’ was set to 50 to increase the running speed. Then, ‘imr imrcall’ (imr imrcall –o imr.sdi –p 50 dangshansuli.fasta imr.bam) was run to detect variants from short reads. The input files were the indexed reference genome fasta file and the specific BAM file. The ‘-p’ for this step was also set to 50.

The command ‘denom soapinterface’ was used to run DENOM by switching on SOAPdenovo, which creates soap4denom.contig, soap4denom.bam and soap4denom.sdi files (denom soapinterface –o denom.sdi –p 50 configfile). Before running, SOAPdenovo was installed on the server. The input file was configfile. The option ‘-p’ was set to 50 to increase the running speed.

The final step was ‘mcmerge dscmp’ to merge the imr.sdi and denom.sdi files (mcmerge dscmp –o merge.sdi dangshansuli.fasta imr.sdi denom.sdi). The input files included the indexed reference genome fasta file, the imr.sdi file and the denom.sdi file. The output file was the merge.sdi file, which contained the chromosome ID, position, length, reference base, consensus base and the quality value.

#### Platypus [35]

Platypus v0.8.1 is a Python, Cython and C package, which has the option ‘assemble’ for detecting SVs based on assembly. It can detect SNPs, insertions, deletions and MNPs. However, it can only detect variants that are less than 10 kb in length. The option ‘assembleBadReads’ was set to 1 for using filtered low quality reads for local assembly. The option ‘assembleBrokenPairs’ was set to 1 for using broken read pairs for local assembly (python Platypus.py --refFile dangshansuli.fasta –bamFile yali.bam –nCPU 50 –assemble 1 –assemblerKmerSize 85). The output file was a VCF file containing the chromosome ID, position, length, reference base, consensus base and the quality value.

#### DELLY [27]

DELLY v0.7.7 is a C++ application and an integrative program for SV discovery that combines short-range and long-range paired-end mapping and split-read analysis. The command ‘delly call’ was used to discover and genotype SVs. The input files consisted of the indexed reference fasta file and the ‘Yali’ sorted BAM file. The generic option ‘-t’ can be changed to detect other types of SVs. Here, ‘DEL’ (deletion), ‘DUP’ (duplication), ‘INV’ (inversion) and ‘INS’ (insertion) were selected to detect different types of SVs (delly call –t DEL -g dangshansuli.fasta yalisortrmdup.bam). The output was in BCF format. Then, Bcftools was used to convert BCF format into VCF format. The output file contained the SV type, the chromosome ID, the SV position, the reference sequence, the alteration, the quality, the filter and other SV information.

#### Lumpy [28]

Lumpy v0.2.13 is a C++ software package that integrates multiple SV signals, such as split reads, read pairs and read depth. ‘lumpyexpress’ in this package was used to detect SVs for standard analyses. Before detecting SVs, the BAM files *.bam, *.splitters.bam and *.discordants.bam were obtained using the BWA-MEM alignment in speedseq [48] (speedseq align -R “@RG\tID:id\tSM:sample\tLB:lib” dangshansuli.fasta yali.read_1.fq yali.read_2.fq). The output contained important information such as the type and size of SV, the chromosome ID, and the SV length (lumpyexpress –B yali.bam –S yali_splitters.bam –D yali.discordants.bam –o yali.output). Lumpy can predict four types of SVs: deletions, inversions, duplications and translocations.

#### MetaSV [30]

MetaSV v0.5.2 is a Python package that uses multiple algorithms: Pindel [25], BreakDancer [33], and Lumpy [28]. The reference, the BAM file, and the outputs of Pindel, BreakDancer and Lumpy were regarded as the input files for MetaSV (run_metasv.py --reference dangshansuli.fasta --breakdancer_native breakdancer.out --pindel_native pindel_D pindel_SI pindel_TD pindel_INV -- lumpy_vcf yali.vcf --bam yali.bam --outdir. /out --disable_assembly). We only merged the outputs of three SV detectors without performing further soft-clip based analysis or local assembly.

#### Sniffles [16]

Sniffles v1.0.11 is a C++ software package that detects SVs based on the split-read algorithm. Sniffles can detect SVs using PacBio or Oxford Nanopore sequencing data. The input file was the BAM file generated from the BWA (‘bwa mem -x’) or NGMLR aligner. Default parameters were used (sniffles –m yalisortrmdup.bam –v yali.vcf). The output file was in ‘vcf’ format and included chromosome, position, SV type, quality and other information.

#### SVIM [32]

SVIM v1.2.0 is a Python package that can detect five types of SVs (deletions, insertions, inversions, break ends, and duplications) using long reads from PacBio or Oxford Nanopore sequencing technology. The input file can be a fastq file or BAM file. To obtain the output file faster, we used the BAM file generated from the NGMLR aligner and ran the command (svim alignment yalisortrmdup.bam dangshansuli.fasta). The output directory ‘candidates’ contained BED format files of each SV type, and each BED file included the chromosome, start coordinate, end coordinate, SV type, score and other information.

### GO and KEGG analysis

Based on the GO annotation information for all genes in the pear genome, WEGO (http://wego.genomics.org.cn/) was used to perform GO analysis. KEGG analysis of genes was done using the website http://www.genome.jp/kegg/.

### Computing resources

Trimming, mapping and SV detection were performed on a server machine equipped with four 2.4GHz Intel® Xeon® 6 CPUs, with 18 cores within each CPU, and 2 TB of RAM. The operating system was CentOS release 6.8.

### Validation of SVs detected using NGS data and long-read sequencing data

The SV calling tools are designed to maximize sensitivity. For example, to identify as many variants as possible in our data, we allowed many false positive calls due to artifacts from the sequencing process. Therefore, before validating the SVs, the SVs called by different software needed to be filtered. For Pindel, we selected the SVs with ‘Support > 5’. For DELLY, Lumpy and MetaSV, SVs with no ‘LowQual’ were selected, and for BreakDancer, SVs with scores over 60 were selected. For SVIM, SVs with a quality of ‘PASS’ were selected, and for IMR/DENOM, we removed the SNPs. Next, the IGV was used to visualize the SVs [49–51]. Specifically, the reference ‘Dangshansuli’ pear genome was loaded as the reference genome sequence, and the BAM file of ‘Yali’ was loaded to visually confirm the presence of the identified deletions and insertions.

## Supplementary information

**Additional file 1.** GO analysis of 264 genes within SVs commonly identified by five SV callers.

**Additional file 2.** KEGG analysis of 264 genes within SVs commonly identified by five SV callers.

**Additional file 3: Figure S1.** The number of genes within SVs detected by software packages using NGS data (a) and long-read sequencing data (b).

**Additional file 4.** GO analysis of 403 genes within SVs commonly identified by three SV callers using NGS data.

**Additional file 5.** KEGG analysis of 403 genes within SVs commonly identified by three SV callers using NGS data.

**Additional file 6.** GO analysis of 4495 genes within SVs commonly identified by two SV callers using long-read sequencing data.

**Additional file 7.** KEGG analysis of 4495 genes within SVs commonly identified by two SV callers using long-read sequencing data.

**Additional file 8.** Verification of SVs in ‘Yali’ through comparisons with the ‘Dangshansuli’ reference genome.

## Data Availability

All raw sequence data generated in this study are deposited in NCBI under BioProject accession number: PRJNA574796. All other supporting data are included as additional files.

## Abbreviations

BAM file: Binary version of SAM file
BCF: Binary Counterpart Data
GO: Gene Ontology
InDel: Insertion and deletion
KEGG: Kyoto Encyclopedia of Genes and Genomes
MNP: Multiple nucleotide polymorphism
NGS: Next-generation sequencing
SAM: Sequence alignment/map format
SNP: Single nucleotide polymorphism
SV: Structural variant
VCF: Variant Call Format
