# Supplementary figures and images for: Comparison of multiple algorithms to reliably detect structural variants in pears
Source: BMC Genomics. 2020 Jan 20;21:61. doi: 10.1186/s12864-020-6455-x (PMC6972009; doi:10.1186/s12864-020-6455-x)

a

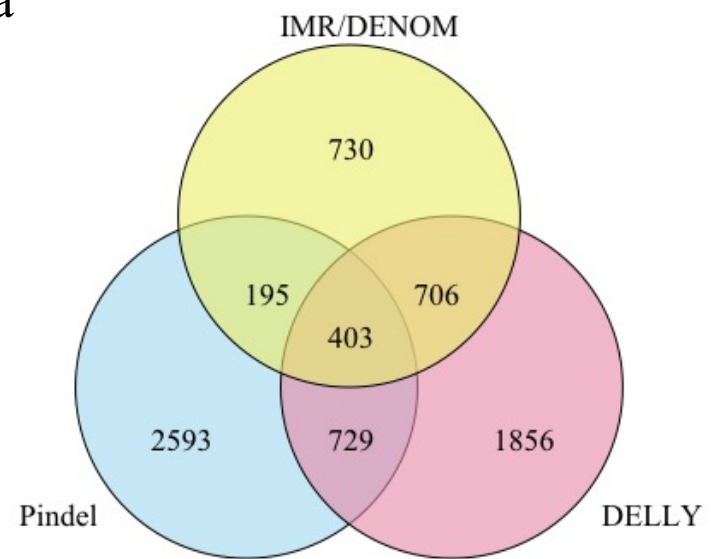

b

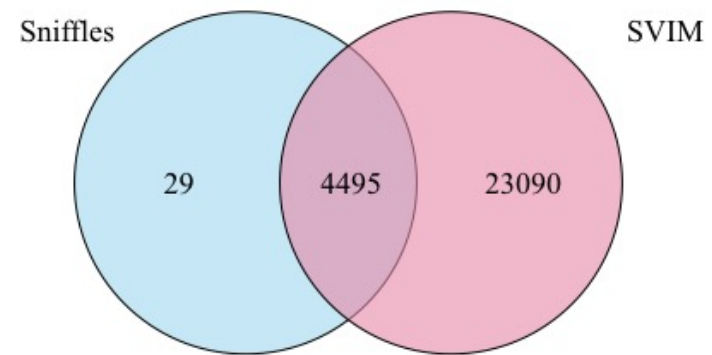

Supplement: Supplementary file 3 — Additional file 3: Figure S1. The number of genes within SVs detected by software packages using NGS data (a) and long-read sequencing data (b). [file 12864_2020_6455_MOESM3_ESM.pdf]
