# Supplementary material for: Comparison of multiple algorithms to reliably detect structural variants in pears
Source: BMC Genomics. 2020 Jan 20;21:61. doi: 10.1186/s12864-020-6455-x (PMC6972009; doi:10.1186/s12864-020-6455-x)
Supplement: Supplementary file 8 — Additional file 8. Verification of SVs in ‘Yali’ through comparisons with the ‘Dangshansuli’ reference genome. [file 12864_2020_6455_MOESM8_ESM.docx]

Table S1: Verification of SVs in ‘Yali’ through comparisons with the ‘Dangshansuli’ reference genome.

|  | combination | 50-100bp | 100-200bp | 200-300bp | 300-400bp | 400-500bp |
| --- | --- | --- | --- | --- | --- | --- |
| NGS_data | Pindel | 75.0% | 68.8% | 58.8% | 53.8% | 33.3% |
|  | BreakDancer | - | - | 54.6% | 50.0% | 70.0% |
|  | Platypus | 64.5% | 78.9% | 53.8% | 62.9% | 71.4% |
|  | IMR/DENOM | 76.4% | 76.5% | 82.3% | 87.5% | 80.0% |
|  | DELLY | 78.1% | 58.8% | 64.7% | 52.9% | 62.5% |
|  | lumpy | 75.0% | 45.0% | 68.7% | 50.0% | 62.5% |
|  | MetaSV | 82.3% | 66.6% | 70.5% | 82.3% | 50.0% |
|  | Pindel_DELLY | 54.2% | 67.8% | 68.8% | 45.4% | 30.0% |
|  | Pindel_BrekDancer | 29.4% | 47.0% | 56.3% | 62.5% | 53.3% |
|  | DELLY_BreakDancer | 85.0% | 63.6% | 72.7% | 53.3% | 87.5% |
|  | Pindel_IMR/DENOM | 82.2% | 75.0% | 60.0% | 80.0% | 50.0% |
|  | BreakDancer_IMR/DENOM | 80.0% | 70.0% | 80.0% | 84.2% | 76.2% |
|  | DELLY_IMR/DENOM | 85.0% | 79.2% | 85.0% | 85.7% | - |
|  | MetaSV_IMR/DENOM | 95.0% | 100.0% | 100.0% | 100.0% | 75.0% |
|  | Pindel_DELLY_BreakDancer | 74.0% | 56.7% | 50.0% | 16.7% | - |
|  | Pindel_IMR/DENOM_BreakDancer | 77.5% | 75.0% | - | - | - |
|  | Pindel_DELLY_IMR/DENOM | 84.9% | 50.0% | - | - | - |
|  | DELLY_IMR/DENOM_BreakDancer | 70.0% | 70.5% | 87.5% | 86.7% | 76.9% |
| Pacbio_data | Sniffles | 94.1% | 93.0% | 95.0% | 100.0% | 94.0% |
|  | SVIM | 82.3% | 76.5% | 76.5% | 82.3% | 68.8% |
|  | SVIM_sniffles | 94.1% | 100.0% | 94.1% | 94.1% | 100.0% |
